# Template based precursor route for the synthesis of CuInSe_2_ nanorod arrays for potential solar cell applications

**DOI:** 10.3762/bjnano.4.98

**Published:** 2013-12-10

**Authors:** Mikhail Pashchanka, Jonas Bang, Niklas S A Gora, Ildiko Balog, Rudolf C Hoffmann, Jörg J Schneider

**Affiliations:** 1 Fachbereich Chemie, Eduard-Zintl-Institut, Fachgebiet Anorganische Chemie, Technische Universität Darmstadt, Petersenstraße 18, 64287 Darmstadt, Germany

**Keywords:** CIS, light absorption, nanocasting, nanorod arrays, precursor synthesis

## Abstract

Polycrystalline CuInSe_2_ (CISe) nanorods are promising for the fabrication of highly efficient active layers in solar cells. In this work we report on a nanocasting approach, which uses track-etched polycarbonate films as hard templates for obtaining three-dimensionally (3D) arranged CISe nanorod arrays. Copper and indium ketoacidoximato complexes and selenourea were employed as molecular precursors. Arrays of parallel isolated cylindrical pores of 100 nm nominal diameter and 5 μm length were used for the infiltration of the precursor solution under inert atmosphere, followed by drying, thermal conversion into a preceramic ‘green body’, a subsequent dissolution of the template, and a final thermal treatment at 450 °C. The nanorods that where synthesised in this way have dimensions equal to the pore sizes of the template. Investigation of the CuInSe_2_ nanorod samples by spectroscopic and diffraction methods confirmed a high purity and crystallinity, and a stoichiometric composition of the CISe ternary semiconductor compound.

## Introduction

Polycrystalline heterojunction solar cells with a columnar morphology of the photovoltaic active layer that are based on the chalcopyrite compound CuInSe_2_ (CISe) have been intensively studied [1]. The basic advantages of CuInSe_2_ as a light absorbing material are its high photovoltaic efficiency and the stability of its properties over time. Apparently, the conversion efficiency can be improved by the increase of the effective absorbing area, and this gave rise to the study of thin film solar cells that are composed of finely divided nanocrystals [2–3]. In this respect, quasi one dimensional (Q1D) nanostructures, such as nanorods and nanowires, have received considerable interest because of their unique ability for independent adjustment of light absorption (by nanowire length) and charge separation (by nanowire diameter). Nanowire-based photovoltaic layers will allow the fabrication of low-cost small size energy devices with economical use of materials. Very recently, Schoen et al. reported the VLS synthesis of CuInSe_2_ nanowires (by Cu impregnation of In_2_Se_3_ nanowires) and the construction of a single-nanowire CIS/CdS core–shell device [4]. However, the authors estimated the efficiency of their solar cell to be below 1%, and the construction of a larger scale device with this approach still remains questionable. Earlier, template-based solution precursor routes were demonstrated to be useful as a fully controllable, simple and inexpensive alternative to vacuum techniques that operate in the VLS growth mode. Large arrays of vertically aligned CISe nanowires were fabricated by electrodeposition into porous alumina templates [5–6]. The nanowires were composed of 5 nm grains and had a noticeable spread in diameter values (10–30 or 25–40 nm, depending on the pore size of the used template) and lengths (0.6–5 μm). Interestingly, authors reported a preferential growth in the [112] direction, whereas the template-based method is commonly known for producing nanowires that are composed of smaller and randomly oriented crystal units [7]. In a similar work, Hernández-Pagán et al. switched between p-type Cu-rich and n-type In-rich CISe by changing the electrodeposition potential [8]. Thus, they indirectly confirmed the flexibility of the solution route in preparation of semiconductors with controlled elemental composition.

We recently successfully demonstrated electroless deposition of molecular precursors (Cu- and In-oximato complexes and thiourea) into track-etched polycarbonate templates and the synthesis of stoichiometric ternary CuInS_2_ nanorod arrays [9]. In the present work, we extend our method to the photochemically even more active CuInSe_2_ material and demonstrate the synthesis of uniform polycrystalline CuInSe_2_ nanorod arrays. Selenourea was used as a Se source analogous to thiourea in our previous investigation. The facile precursor method provides many benefits over currently used selenization techniques [10–12], or the impregnation of a third metal cation into a binary selenide compound [4,13]. Firstly, it achieves mixing at the atomic level by forming a solid ‘green body’, in which the elements are present in the correct stoichiometry [14]. Secondly, the method allows to lower the conversion temperature and to reduce the particle size. Thus, the products usually contain small particles of large surface area, which is favourable for visible light absorption. Finally, the precursor solution route does not employ the highly toxic gaseous hydrogen selenide as a Se-source. However, suitable precursors are not always available for a desired functional material, but nevertheless there are already several reports on different single source molecular precursors for chalcopyrite type CuInSe_2_ in the current literature [15–17]. It has to be mentioned that the morphologies accessible by the liquid precursor route are not restricted to only Q1D nanostructures. Such stable ‘inks’ can also find application in printable photovoltaics or film deposition onto various standard substrates, e.g., polycrystalline alumina, low-cost glass or even flexible polymeric films [18–19].

## Experimental

**Scheme 1:**
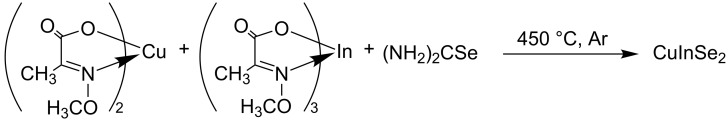
Reaction scheme of the ternary CuInSe_2_ compound obtained by the precursor synthesis method employing Cu(II) and In(III) oximato complexes.

In- and Cu-oximato complexes were synthesised as reported earlier and were used as metal cation sources [20–21]. A solution of Cu-oximato and In-oximato precursors, and selenourea in 1:1:3 molar ratio was prepared in 2-methoxyethanol under inert atmosphere (all steps employing selenourea were performed in an Ar glovebox). It was found during our previous study on the ternary CuInS_2_ system that the stoichiometric 1:1:2 molar ratio of molecular precursors in the solution yields chalcogen-deficient products with a minor content of secondary phases, which can be completely avoided by providing an excess of the chalcogen-containing component [9]. Hence, excess selenourea (1.5 times) was taken for the present work. After adding the solvent to the mixture of precursor powders, the solution gains an intensive dark-brown colour. A porous polycarbonate film (Whatman Nuclepore™ track-etch membrane, nominal pore size 0.1 μm) was immersed into the solution, ultrasonicated for 5 min for gas removal and complete pore infiltration (in a sealed flask under inert gas), and dried at ambient temperature (in the glovebox). After that, the membranes were cleaned from the excess of precursors with a lint-free tissue. The first solidification step of the nanorods (formation of ‘green body’) was performed in a quartz tube under Ar flow at 180 °C for 2 h. The filled templates were then immersed in CH_2_Cl_2_ to remove the polymeric film completely and the obtained powder was dried in air (the solidified green body nanorods were stable in the air conditions). Finally, the dry solid residue was placed in a quartz tube under Ar flow at 450 °C for another 2 h. The temperature at this stage should not exceed 450 °C, since a brick-red deposit (poly-selenide) covered the colder parts of the quartz tube outside the furnace (the entire process is shown in Scheme 1).

The final ceramic CuInSe_2_ nanowires had a deep black colour. The samples were characterised by scanning electron microscopy (SEM) by using a Philips XL-30 FEG electron-scan microscope coupled with an energy-dispersive X-ray (EDX) analyser that was operated at 20–25 kV. The samples were mounted on conductive carbon-rich polymer films and sputtered with a Pt/Pd alloy. Transmission electron microscopy (TEM) images were taken by using a Tecnai G2 F20 microscope operated at 200 kV, the samples were dispersed in ethanol by ultrasonication and then deposited on copper TEM grids. X-ray analysis (XRD) of the species was carried out on a Stoe&Cie StadiP diffractometer in Debye–Scherrer geometry while using Cu Kα_1_ radiation (λ = 1.541 Å) with a Ge(111) monochromator. Raman spectra were recorded from 50 to 2000 cm^−1^ with a micro-Raman HR800 spectrometer (Horiba Jobin Yvon, Bensheim, Germany) while using laser wavelength of 514 nm. For the recording of absorption spectra the remission method was used. With a UV–vis–NIR spectrometer Lambda 900 from Perkin Elmer the remission was recorded and automatically converted to an absorption spectrum via the Kubelka–Munk-relation ((1−*R*)^2^/(2*R*), *R* is the normalized remission).

## Results and Discussion

**Figure 1:**
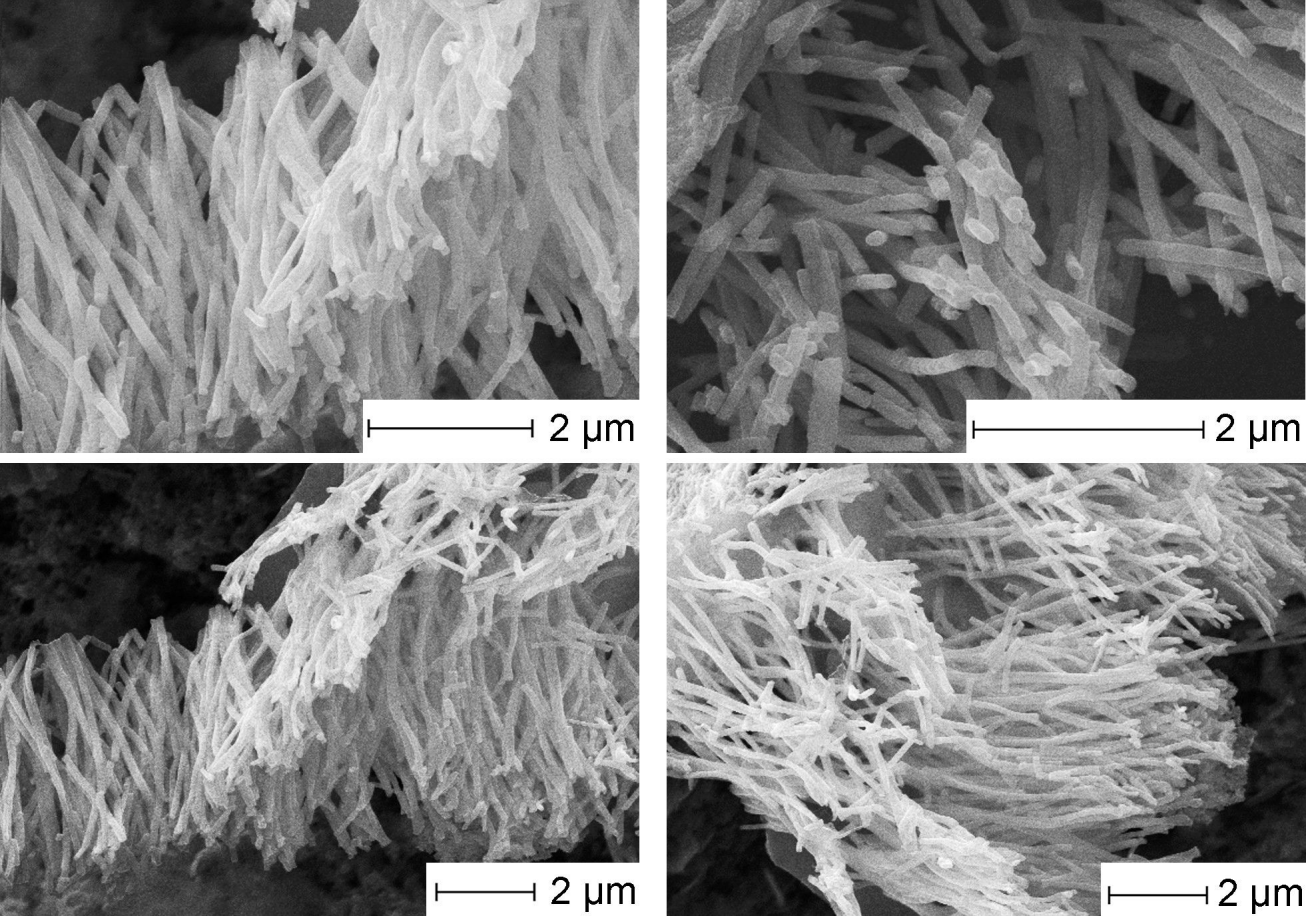
SEM micrographs of CuInSe_2_ nanorod arrays after the final conversion step at 450 °C.

The morphology of the material after the final conversion step was examined by using SEM (Figure 1). The product consisted of separated parallel nanorod bundles. The nanorod diameter was about 100 nm and the length 5 μm, which is in full agreement with the pore sizes of the utilized polycarbonate templates.

**Figure 2:**
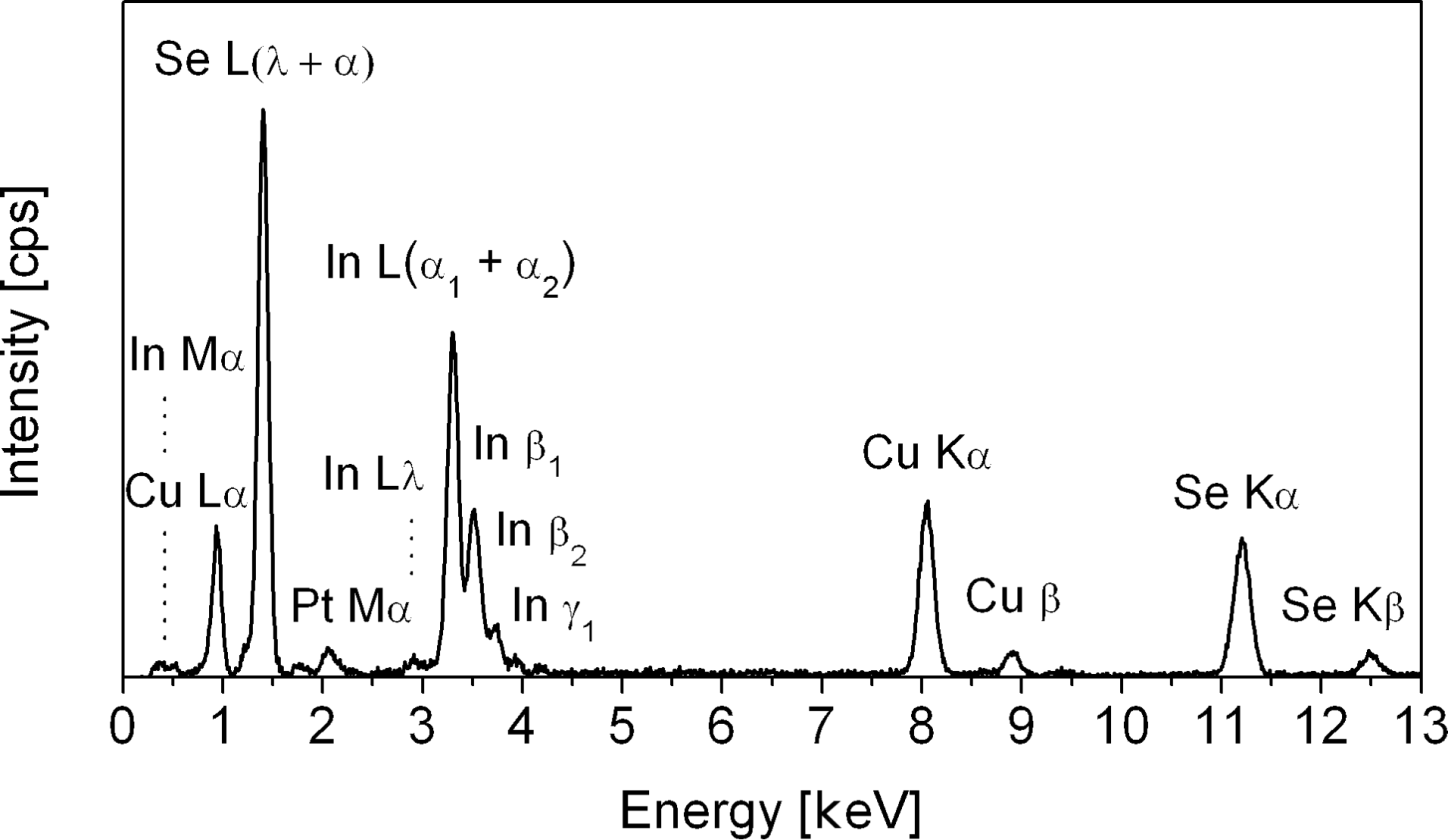
EDX analysis of CuInSe_2_ nanorod arrays; Pt-signal originates from the sputtered Pt/Pd alloy.

The material demonstrated good stability after calcination at 450 °C, all nanorods had a smooth surface and uniform length and showed no cracks along the rod length. The EDX-analysis (Figure 2) showed a high purity of the product, as well as a homogeneous distribution and nearly ideal stoichiometric ratio of the constituting elements. According to the standardless quantification method, the nanorods contain 19.0 wt % of Cu, 36.5 wt % of In, and 44.5 wt % of Se (the theoretical contents are 18.9, 34.1, and 47.0 wt %, correspondingly). The deviation from theoretically calculated values towards indium enriched CuInSe_2_ is practically within the limits of the error of the individual method. The atomic Cu:In ratio is approximately 0.95, and the measured selenium deficiency is only around 2 at %.

**Figure 3:**
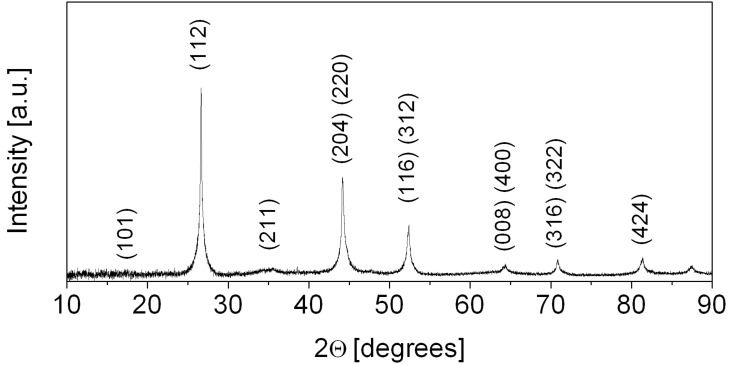
Powder X-ray diffraction pattern of polycrystalline CuInSe_2_ nanorods after final conversion at 450 °C.

Thus, if the formation of secondary phases occurs, only minor concentrations of indium selenide binaries are possible, which nevertheless has to be confirmed by other analysis methods. The purity of the sample was thus further characterised by powder X-ray diffractometry. The XRD analysis (Figure 3) demonstrated no secondary selenide phases or other impurities in the crystalline product. All of the reflections correspond solely to copper indium selenide (JCPDS-file 80-535). All signals are visibly broadened, which is due to the nanocrystallinity of the particles that compose the rod morphology. The mean crystallite size calculated from the most intense (112) peak by using the Scherrer equation is about 30 nm, which is approximately three times smaller than the nanorod diameter.

**Figure 4:**
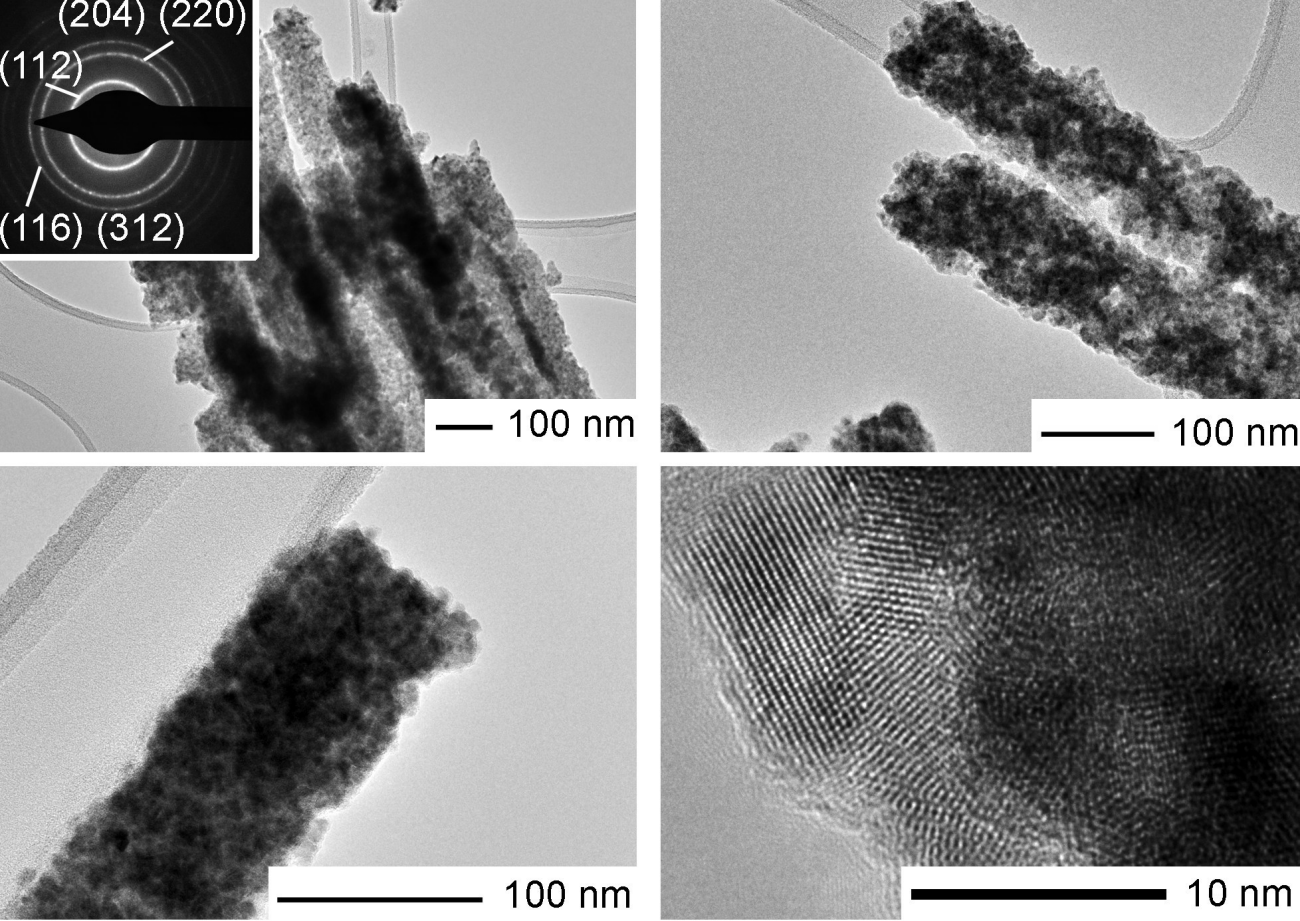
TEM images and SAED micrograph of polycrystalline CuInSe_2_ nanorods.

The polycrystalline nature of the product was also confirmed by TEM and SAED (Figure 4). Diffuse rings in the electron diffraction pattern (see inset in the upper left corner in Figure 4) suggest a random crystallite orientation and no preferential crystal growth direction. At a higher magnification it can be recognized that the rods are composed of nanocrystals of approximately 5–10 nm in size, which is in contrast to the determined mean crystallite size as obtained from the Scherrer equation. However, it can be seen that if one moves across an individual rod, the individual crystallites mostly overlap, thus their exact individual size cannot be determined. Possibly, smaller crystallites are distributed at the surface, and a crystal size enlargement takes place near the rod core, so that the average size equals to 30 nm. It has to be mentioned that the samples for TEM/SAED were dispersed in ethanol by ultrasonication before their deposition onto a copper grid, hence, the nanorod bundles can be partially disassemble and individual nanorods unhinge from the structures.

**Figure 5:**
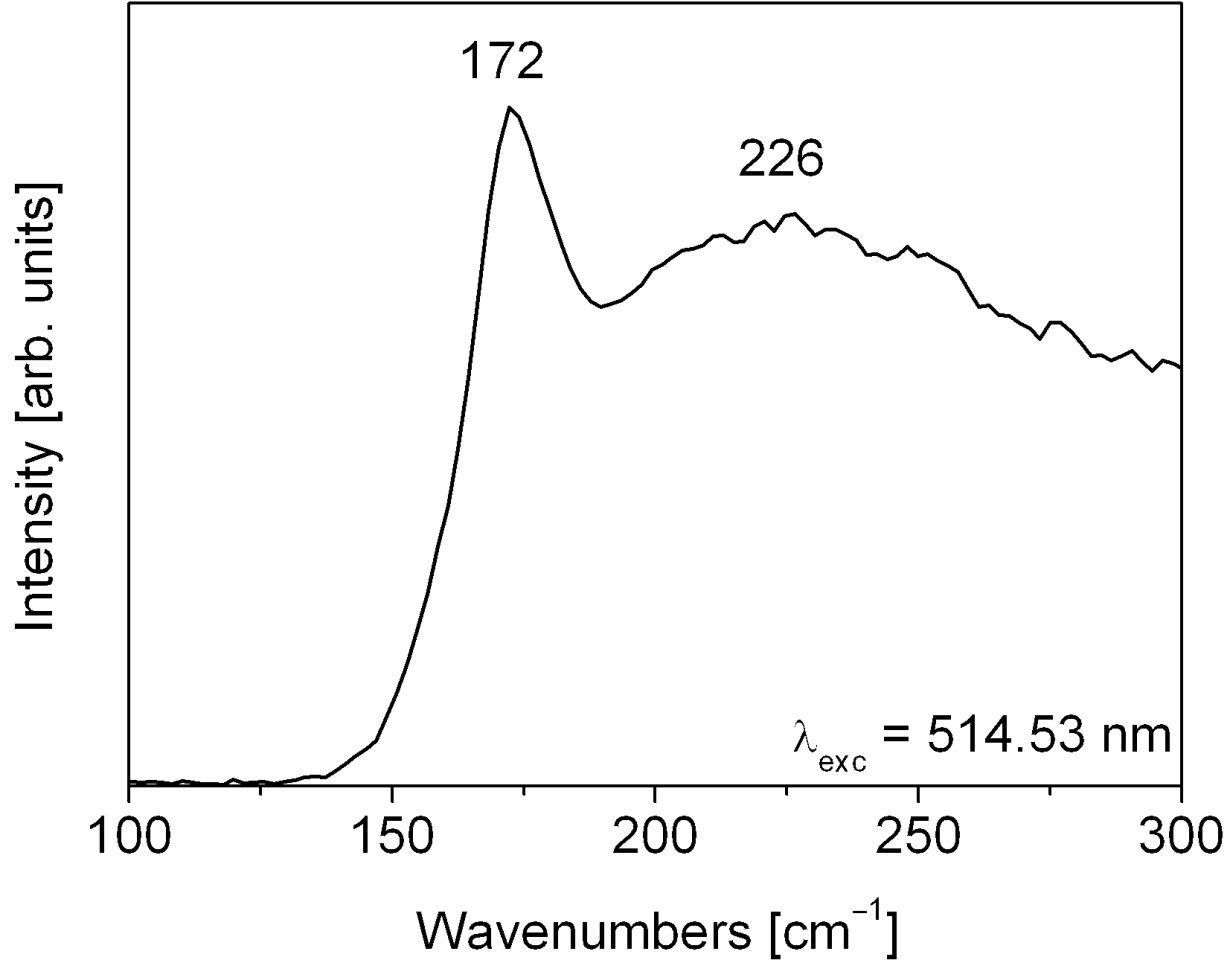
Raman spectrum of CuInSe_2_ nanorod arrays.

The phase and purity of the CuInSe_2_ material were further confirmed by Raman scattering (a typical micro Raman spectrum is presented in Figure 5). Copper indium selenide is characterised by a large absorption coefficient, and incident light can reach penetration depths only up to 100–200 nm, which results in a low signal intensity [22–23]. However, the penetration depth corresponds well with the nanorod diameter, and reliable information about the structural properties can be thus obtained. The most intensive peak at 172 cm^−1^ results from the Γ_1_ chalcopyrite phonon mode (selenium anion vibration) [24]. This signal is commonly observed in CuInSe_2_ thin films and nanoparticles, and its intensity is associated with the crystalline quality [25–27]. A moderate shift of this peak to lower wavenumbers may result from structural defects like grain boundaries, and confirms the nanocrystalline composition of the nanorods [27]. The broad structureless signal centred at 226 cm^−1^ is also in good agreement with previous studies of CuInSe_2_ lattice dynamics (multiple signals in the range between 198 and 260 cm^−1^) [24,28–29]. It is also important to note, that there are no prominent peaks with Raman shifts corresponding to secondary indium or copper selenides, which denotes a higher homogeneity of the material than can be achieved by selenisation of metallic precursors or the electrodeposition technique [11,23].

**Figure 6:**
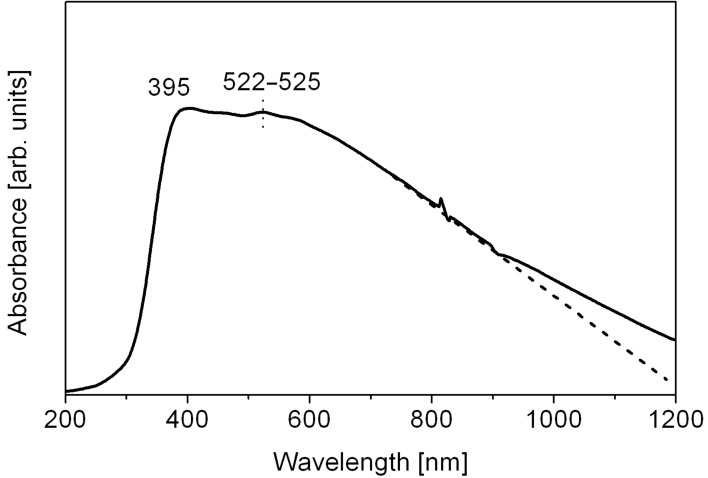
The absorption spectrum of polycrystalline CuInSe_2_ nanorods.

To characterize the light absorbing properties of nanorods, the UV–vis–NIR spectrometry was employed (Figure 6; note that the absorption is given in arbitrary units). The absorption starts in the UV region at λ = 210–220 nm and reaches the maximum at 395 nm. The maximal absorption covers practically the whole visible region and begins to decrease slowly at approximately 575–590 nm; a little broad peak is observed at 522–525 nm, which corresponds to 2.36–2.38 eV. The artifact between 800 and 900 nm is due to the grating change of the two monochromators, which has gratings for each the NIR- and for the UV–vis-range. Some distinctive features can be observed in comparison with earlier reports on nanostructured CISe materials. As a rule, CuInSe_2_ CISe nanostructures of different shapes demonstrate a high absorption in the UV region, a maximum peak at 440–540 nm (which corresponds to a band gap of 2.8–2.3 eV) within the visible range, and the absorption spectrum threshold at around 550–900 nm [25,30–31]. In contrast to these examples, our material shows only a moderate absorption in the near ultraviolet region, and practically does not absorb at wavelengths of 200–300 nm. Instead, it shows superior performance compared to previously reported CISe nanomaterials in the whole visible region, with a remarkable value of absorption extending to the NIR region as well. This could be a benefit for a potential application in solar cells, since the amount of UV light is not constant during the course of the day. Distinctive in the spectrum in Figure 6 is the abnormally high threshold (where the dashed line in Figure 6 meets the wavelength axis); it surely goes beyond the range of measured wavelengths, but even at 1200 nm it would correspond to an absorption band gap of 1.03 eV, which perfectly matches the red edge of the solar spectrum (0.8–1.1 eV). The reason for this ‘red shift’ according to the previous reports on CISe nanostructures is not entirely clear, because the nanorods are composed of small 10–30 nm particles (as follows from the XRD and TEM analysis data). The UV–vis–NIR absorption results may count in favour of the mean crystallite size calculated by Scherrer formula (30 nm), and indirectly confirm that smaller 10 nm nanoparticles, which are visible in TEM images, are mainly distributed on nanorod surface and in the surface region.

## Conclusions

In conclusion, we successfully synthesised aligned arrays of CuInSe_2_, CISe, nanorods with controllable composition and high purity and homogeneity of the material. The CISe nanorods are composed of smaller randomly oriented nanocrystals of 30 nm mean size. As follows from the diffraction analysis and TEM examination, smaller 5–10 nm crystallites are mainly concentrated on the surface and in the surface region of the rods, while the rod core presumably consists of larger nanocrystals. The crystalline chalcopyrite phase was also confirmed by Raman spectroscopy, which suggested that there are no secondary binary selenides in the synthesised ternary compound. The light absorbing properties showed some distinctive characteristics in comparison with previously reported CISe nanomaterials; the nanorods moderately absorb in the near-UV region, and a good level of absorption covers the whole visible range and a part of the near infrared diapason as well (with a threshold that corresponds to a bandgap energy of 1.03 eV). A future challenge would be the incorporation of the 3D aligned CISe nanorod arrays as absorber material in a solar cell. Obviously, one of the main challenges towards this end is to achieve a transfer of the aligned nanorods onto a conductive substrate. A possible way to achieve that could be a direct placement of the polycarbonate template after infiltration with the precursor molecules onto a Mo-coated glass substrate, followed by thermal conversion into CuInSe_2_ nanorod arrays. Another possibility could be a direct deposition of the as-prepared CuInSe_2_ nanorods from dispersions in organic solvents directly onto the appropriate substrate. Both routes are currently studied in our laboratories.
